# Oral Health Beliefs, Attitudes, and Practices of South Asian Migrants: A Systematic Review

**DOI:** 10.3390/ijerph16111952

**Published:** 2019-06-01

**Authors:** Mehak Batra, Sabrina Gupta, Bircan Erbas

**Affiliations:** Department of Public Health, School of Psychology and Public Health, La Trobe University, Bundoora, VIC 3086, Australia; mehakcd@gmail.com (M.B.); S.Gupta@latrobe.edu.au (S.G.)

**Keywords:** migrants, South Asians, knowledge, behaviors, oral health

## Abstract

Oral health is a burden among all populations and is linked with major chronic diseases such as cardiovascular diseases. Migrants, in particular South Asians, have poor oral health which requires further understanding to better inform oral health interventions by targeting specific aspects of this heterogenous South Asian population. This review is undertaken to systematically synthesize the evidence of oral health understandings, knowledge, attitudes, beliefs, practices, and behaviors of South Asian migrants residing in high-income countries. A comprehensive systematic search of seven electronic databases and hand-searching for peer-reviewed studies was conducted. All study designs were included, and quality assessment conducted. Of the 1614 records identified, 17 were included for synthesis and 12 were quantitative in design. These studies were primarily conducted in the UK, USA, Canada, and Europe. South Asian migrants had inadequate oral health knowledge, attitudes, and practices—influenced by culture, social norms, and religiosity. In the absence of symptoms, preventive oral hygiene practices were limited. Barriers to access varied with country of origin; from lack of trust in dentists and treatment cost in studies with India as the country of origin, to religiosity, among poorer nations such as Bangladesh. Fewer studies focused on recent arrivals from Bhutan or the Maldives. Culturally and socially appropriate strategies must be developed to target oral health issues and a “one-size” fits all approach will be ineffective in addressing the needs of South Asian migrants.

## 1. Introduction

Dental diseases such as dental caries, periodontal diseases, and oral cancer have increased in prevalence globally by an average of 45.6% since 1990, in parallel with major non-communicable diseases (25.0%) [1]. Poor oral health impacts substantially on daily activities due to the associated pain and suffering, and in the long term is a substantial health and financial burden [1]. Delayed detection in younger people may result in complications later in life [2]. In early childhood, dietary and hygiene habits are influenced by their caregivers and oral health literacy is paramount. Mattos and colleagues [3] recommended that dentists treating children should encourage their caregivers to receive dental care and education on healthy habits. Impacts of poor oral health are not limited to the mouth, as it is not an isolated organ, but to overall general health [4]. Dental infections and conditions resulting from dental surgeries could all result in a compromised immune system due to the spread of bacterial infections in the organism [5]. Furthermore, poor oral hygiene resulting in oral or dental infections have been established as independent important risk factors for chronic diseases, such as cardiovascular diseases [5]. Notwithstanding this knowledge, oral health is one of the most ignored areas of healthcare globally and often is low on the list of priorities in light of other more chronic diseases that cause a greater burden with higher mortality, with the exception of oral cancer.

Oral health still remains an issue in many developed countries [6] and it is an underexplored issue in many developing countries, particularly South Asian countries including Bhutan, Bangladesh, India, Pakistan, the Maldives, Myanmar, Nepal, and Sri Lanka [7]. Oral health literacy is low and self-taught, and unqualified and unregulated healthcare providers often provide treatments under primitive and hazardous conditions [6]. Furthermore, there is a lack of basic dental care aids such as toothpaste containing fluoride and traditional approaches to dental care are maintained [6]. 

Migrants from developing countries who migrate to high-income countries such as the UK, USA, Canada, and Australia are also known to be at risk of poor oral health [8,9,10]. South Asian populations represent a good proportion of the migrants in high-income countries. For example, in the USA there are over 4.3 million South Asians [11] and in Australia, South Asians are presently the largest incoming migrant population [12]. This large-scale migration poses significant challenges for dental healthcare systems in the recipient countries as the settlers bring their own oral health knowledge, beliefs, and attitudes which are usually based on their unique culture, ethnicity, linguistic profile, and past experiences with the healthcare system in their home countries. In addition, navigating the healthcare system in the host country, as well as facing financial constraints, can act as potential barriers to accessing services [9]. For example, in one study it was found that newly arrived migrants from India and Bangladesh in the UK had limited or no experience of any dental care [11]. In another UK-based study [13], there was much heterogeneity with Indians having better oral health-related behaviors compared with migrants from Bangladesh and Pakistan. It was suggested that the difference may have been due to socioeconomic background and/or length of stay in the UK. In another study, Riggs et al. [14] reported that female Pakistani migrants in Australia had a lower uptake of dental services, particularly those pertaining to preventive dental services. 

Numerous studies [14,15,16] have focused on oral health knowledge, attitudes, and practices of South Asian migrants in high-income countries, yet no systematic review has been done to synthesize these findings which can be used to better inform health interventions by targeting specific aspects of the heterogenous South Asian community. Therefore, in this review we sought to systematically synthesize the evidence on oral health understandings, knowledge, attitudes, beliefs, practices, and behaviors of South Asian migrants residing in high-income countries. By identifying existing gaps in knowledge, it may highlight the need for additional studies to target particular areas for interventions. This is an emerging public health issue as a result of the continually high levels of migration from these South Asian countries. 

## 2. Materials and Methods 

This systematic review was conducted with the development of a protocol by the researchers, followed by an electronic literature search and use of the Preferred reporting items for systematic reviews and meta-analysis (PRISMA) guideline [17] as the basis for reporting the findings (Table A1). The protocol for this review was not registered. 

### 2.1. Eligibility Criteria

Inclusion criteria were studies that included South Asian (those from Indian, Sri Lankan, Pakistani, Bhutanese, Nepalese, Bangladeshi, Maldivian, or Afghani backgrounds) adult migrants aged 18 years or over and residing in high-income countries. Additionally, the studies should have explored at least one or more oral health-related knowledge, practices, behaviors, attitudes, beliefs or understandings. There was no limit on the study design. Quantitative studies of clinical outcomes detailing information on South Asian migrants or mixed studies (both quantitative and qualitative) were also eligible. 

### 2.2. Search Strategy 

M.B. and S.G. participated in the literature search, study selection process, and extraction of the studies. Disagreements were resolved by discussion with B.E. The abstracts of all identified papers were reviewed for initial inclusion by researcher’s M.B. and S.G.; then full papers were read by two researchers (M.B. and B.E.) to see if all inclusion criteria were met. 

Publications on the oral health of South Asian migrants were searched between December 2018 and January 2019 through seven electronic databases (CINHAL, Medline, Embase, ProQuest Central, Web of Science, Scopus, and Google Scholar (first 10 pages)). Combinations of keywords and medical subject headings (MeSH) were used (Table 1). Articles were shortlisted if they were in English and any combination of words appeared.

### 2.3. Data Extraction

Data were extracted from all included articles in a standardized manner. Data extracted from each study included: author, year of publication, study design (type of study whether qualitative or quantitative); study population (and further which countries were included as South Asians); country where the study was conducted; number and age of the participants; research methodology used; and the findings from the South Asian groups studied.

### 2.4. Assessment of Quality

Two researchers (M.B. and S.G.) conducted a quality assessment of all included studies using two validated quality assessment tools [18,19]. The tool used for qualitative studies [18] included a description of the research design; description of sampling method; types and appropriateness of data collection methods in each study; whether all the relevant data has been included and whether each study had considered triangulation for the data; and finally the credibility of the findings and whether each study had taken potential bias in account. Similarly, the tool used for the quantitative studies [19] included questions around the research hypothesis, sampling methods and sample size, analysis, and the 5validity of each study included. 

## 3. Results

**Figure 1:**
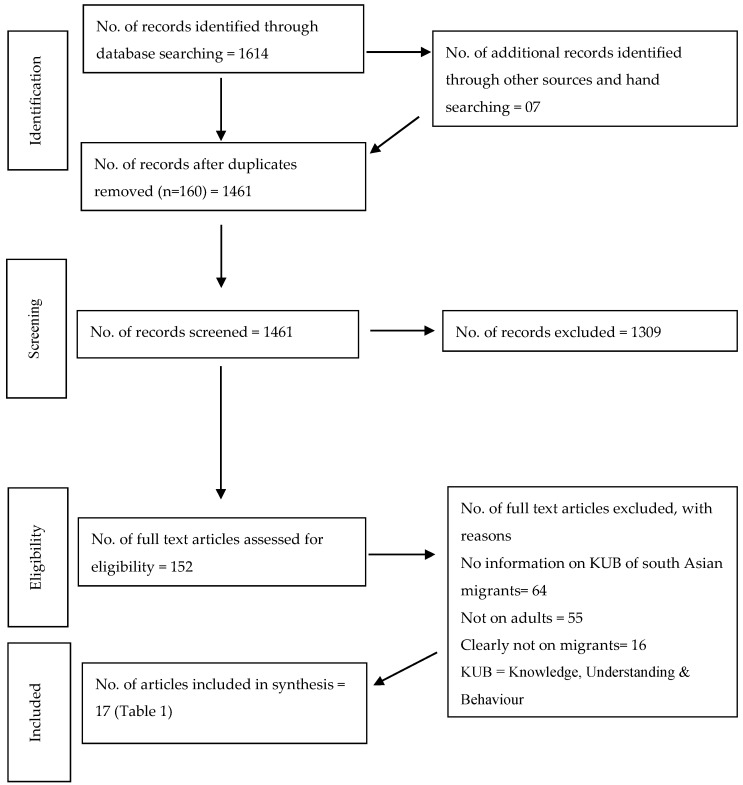
Preferred reporting items for systematic reviews and meta-analysis (PRISMA) flow chart of the study selection process [17].

The comprehensive search resulted in 1461 peer-reviewed scientific articles after duplicates were removed (Figure 1). Of these, 1309 were irrelevant and removed following screening of titles and abstracts. A considerable number of these excluded papers were not relevant to this review because they had no information on oral health for South Asian migrants, were studies conducted in the home countries of South Asians, were review articles or assessed outcomes such as cancer or Qol (quality of life) studies. Finally, 152 full-text articles were assessed and 135 were again excluded as they did not focus on adult populations or did not assess the relevant research question about oral health knowledge, beliefs, behaviors, understandings, and attitudes in the study population (South Asian adult migrants). In total 17 papers were included in this review: 12 quantitative studies and 5 qualitative studies. 

### 3.1. Quality Assessment 

Findings from the quality assessments are presented in Table A2 and Table A3. All of the 12 quantitative studies scored highly except for one study [20], which was due to lack of rigor, inappropriate statistical analysis, and lack of control for potential bias. In three studies [8,16,21] the strata analysis by Bangladeshi [8], Pakistani [16], and Bhutanese [21] was undertaken; however, the sample size was inadequate which limited our comparative understanding of these groups. In addition, a study [9] employed a predictor model for analysis however, the sample size was small which limited the inference concluded from the study. Few studies [21,22] were descriptive in nature, so questions regarding methods used for analysis or minimizing bias were not applicable. All the qualitative studies were of high value. However, the studies scored low for the sample size justification, as data saturation was not discussed in any of the studies. Triangulation of data was only performed and discussed in one study [23], which limited the scoring based on the tool [18].

### 3.2. Synthesis of Qualitative Studies

#### 3.2.1. Design and Setting

Five studies were included in this synthesis (Table 2). Three [23,24,25] were conducted in the UK and two [14,15] were in Australia. Of the two Australian studies, Riggs et al. [14] included Pakistani migrants as one of their ethnic groups and Lamb et al. [15] included Afghani refugees (please note we have used terms to define populations as mentioned by the authors of the included studies). Of the three UK studies, two [23,25] had Indian migrants as one of their common study population groups and the third study [24] had only Bangladeshi and Pakistani migrants representing South Asians. Only two studies considered females [14,24]. Studies used focus groups [23,24,25], in-depth interviews [15] or a combination of both designs [14]. 

#### 3.2.2. Oral Health Knowledge 

Oral health knowledge of South Asian migrants was discussed in three studies [14,15,23,24,25]. William & Gelbier [24] reported the participants (Pakistani & Bangladeshi migrants in the UK) mentioned they lacked oral health knowledge and that they were keen on seeking more information about availability of dental services, treatment options and prevention measures. On the other hand, participants in Riggs et al. [14] and Afghani refugees in Australia [15], discussed and demonstrated that they had knowledge about the importance of dental care services for maintaining good oral health and risk factors for dental injury associated with breaking nuts, removing needles and dried foods common cultural practices. 

#### 3.2.3. Oral Health Attitudes and Practices

Lack of trust in a dentist and dental health services was a major concern identified, particularly by women [14,25]. Indian migrant women in a study in the UK [25] thought that dental treatments are intentionally prolonged whereas the Pakistani migrant women, in the same study, believed that mistrust was exacerbated due to language barriers. Pakistani participants in Australia mentioned that they preferred accessing a dentist in their home country suggesting greater trust in dental practitioners in their home country, a better understanding of the health system there, and perhaps greater affordability of services [14]. In general, cost of dental treatments was perceived as higher in the migrant country [14,23,25]. Pakistani mothers in Australia [14] questioned the absence of dental services from subsidized public healthcare and believed that restorative treatments were both mandatory and expensive. Language was perceived to be a barrier with many being unable to articulate their concerns to the care provider [26]. For example, participants in three studies, had stated their lack of language as a barrier in securing dental appointments [14], a dependency on another person to seek dental care [25], and a main reason for expensive or prolonged treatments [24]. Training dental care staff to speak community languages may improve access to dental care. Oral health behaviors such as rinsing and washing the mouth before every prayer (3–5 times a day), and using ‘miswak’, among Afghani refugees [15], were influenced by their religion (Islam). In three studies, in line with their cultural values, a preference for a female dentist was mentioned by Pakistani women [24,25] and Bangladeshi women [23], whereas gender was not a major issue for Indians of both sexes [23]. Lack of cultural knowledge by the dentist was also believed to be a barrier [25]. Oral health behavior was also influenced by one’s past experiences in accessing dental care [24]. Long waiting lists, unavailability of care at the time of the need, waiting room environments, and poor engagement with administrative staff at care facilities were considered as past experiences hindering current experiences. 

### 3.3. Synthesis of Quantitative Studies

#### 3.3.1. Design and Setting

Of 12 quantitative studies (Table 3), six were conducted in the UK [8,10,13,20,22,27], three in the USA [16,28,29], one in Canada [21], one in Norway [9], and one in Singapore [30]. In nearly all of the studies from the UK, ‘Indian’ ethnicity was the majority study population group representing South Asians with the exception of one study that included only ‘Bangladeshi’ migrants [10]. A Norwegian study focused on ‘Pakistani’ migrants only [9], and a Canadian study focused only on ‘Bhutanese’ migrants [21]. Two studies focused only on women; one study focused on 231 Indian women [27] and the other on 246 Bangladeshi women [10]. Methods for assessing the relevant constructs/concepts such as knowledge, attitude, and practices in all the included quantitative studies have been summarized in Table 3.

#### 3.3.2. Oral Health Knowledge 

Four studies [9,22,28,30] reported some data on oral health knowledge of South Asian migrants. Selikowitz and Holst reported Pakistani migrants in Norway had a high knowledge (64.5%) about the etiology of dental caries [9]. Similarly Asian Indians in the USA scored high on oral health knowledge [28]. In general, there was a lot of emphasis on tooth brushing and regular dental check-ups. However, there was little awareness about the importance of fluoride and sealants in dental treatments. Indian migrants in Singapore, demonstrated a higher knowledge of the importance of brushing and regular dental care compared to their awareness of flossing and dental sealants in prevention of caries and gum disease [30]. Only 32% of Indian migrants in Scotland [22] were aware of the significance of regular brushing against dental caries, but a vast majority of this population [22] had not completed their secondary schooling (62%) and 31% had language problems. 

#### 3.3.3. Oral Health Attitude

Oral health attitudes were assessed in two studies [22,28]. A large proportion of Asian Indians in the USA [28] scored a higher oral health attitude score. Whereas 31% of Indian migrants in the UK [22] considered maintenance of oral health as a religious custom and social acceptability in relation to aesthetics. The population was aware of the importance of natural teeth, however, a significant percentage (61%) of the population would only intend to access dental care in the presence of symptoms (i.e., pain). Asian dentists were also preferred in the same study. 

#### 3.3.4. Oral Health Behaviors/Practices

Tooth brushing, and its frequency have been documented in eight studies [8,13,20,21,22,27,28,30], of which three [13,28,30] suggested tooth brushing as a widespread practice among the South Asian migrant community. However, frequency of tooth brushing varied. In one study [20], a higher number of Pakistani participants compared to Indian and Bangladeshi reported no tooth cleaning. Those who were younger were more likely to brush regularly whereas those in older age [27] did not, possibly due to traditional practices and beliefs.

Flossing was recommended along with brushing, yet 95.1% of Bhutanese in Canada [21], 93.1% of Indians in the UK [27], 64.8% of Indians in the USA [28], and 93.7% of Indians in Singapore [28,30] were reported to have never flossed. However, 68% of Indian migrants in another study affirmed rinsing their mouth after every meal [22] in accordance to their religious norms. Some used traditional methods which include finger, chewing stick, soot, and tobacco powder as their oral hygiene aids [10]. Most studies show a lack of attending the dentist [8,9,10,13,16,20,21,22,28,29,30] and in one study a good proportion had not visited a dentist at all [20]. Ethnicity was not a determinant for dental care utilization given that most studies suggested that visiting a dentist in the absence of symptoms was uncommon. 

The impact of socio-demographic and migration variables such as years lived in the host country was an important consideration. Age of uptake of services was assessed in three studies [9,10,29]. The highest frequency of Bangladeshi migrants (39%) in the age group of 35–44 years and Pakistani migrants (66.7%) aged 25–29 years were found to visit a dentist [10]. Whereas in another study [29] adjusted for age, only 60.7% of Asian Indians visited the dentist in 12 months or less.

## 4. Discussion

To our knowledge, this is the first systematic review that presents evidence of the knowledge and information on behaviors, barriers, understandings, and beliefs among South Asian migrant adults and oral health in high-income countries. It is well-recognized that the dental health of migrant communities is worse compared to the host population in high-income countries [31]. Out of 258 million international migrants worldwide, 106 million are of Asian origin, of which South Asia contributes an enormous number, with the of majority males (55%) and median age of 39 years [32]. Therefore, the synthesis of the current literature on adult migrants from South Asia in high-income countries enabled a thorough understanding of issues associated with these at-risk populations and allowed us to identify knowledge gaps for future studies. Most studies were from the UK due to the high levels of migration in the past from South Asian due to the economic attraction and the long-term regional association between these two regions [33]. As per the 2011 census in the UK [34], South Asians form 4.9% of the overall population, which is significant enough to have been studied and researched for better formulations of public health policies in the UK. In general, among all the studies included, optimal oral health knowledge was found low, but most were using outdated/traditional modes of oral hygiene as their oral health practices were influenced by their culture, religious customs, and beliefs. Moreover, access was a major barrier due to being female, lack of trust, language difficulties, general unawareness, and perceived cost of treatments.

Tooth brushing was mostly a popular practice, but flossing was not. Interestingly, traditional methods of cleaning such as finger, chewing stick, soot, and tobacco powder were still highly prevalent among migrant Bangladeshi women [10]. Consistent with other ethnic populations, South Asian migrants mostly visited dentists only in the presence of symptoms when prevention may be too late, and treatment delayed. The concept of regular checks and preservation of teeth was not a major component of their dental health behavior, as many did not perceive oral health as important. There is a need for more studies to be conducted with a focus on dental education including the importance of dental care especially for women of child-bearing age.

A major barrier in accessing care for women was the lack of trust in a dentist [14,25] or dealing with communication issues in articulating problems to dentists [14,23,24,25]. Cost of dental care was generally perceived higher among migrants, irrespective of the host country or ethnicity. The sex of the healthcare provider was also considered as a barrier among Pakistani [24,25] and Bangladeshi women [23]. In most of the studies where the country of origin was India, barriers were predominately lack of trust and cost of dental treatments. From smaller and less affluent regions, such as Bangladesh, religiosity was reported as a key issue to quality dental care.

The observed reliance on traditional methods of cleaning were significantly associated with socio-demographic factors, educational status, and lack of language skills [10]. Moreover, many women included in the studies who never accessed dental care in regions such as the UK, were illiterate, in the age group of 35–44 years, and had no understanding of English. Lack of language skills was a major barrier in assessing dental services by women of other ethnicities as English was not a primary language. Interestingly, few studies focused on South Asians from the Maldives, Sri Lanka, and Nepal and more investigations need to be carried out to better understand the complexity of these issues among other ethnicities with high levels of migration. 

Preference for the same sex dentist among varying ethnicities is common and reflects on typical South Asian culture [35]. Similarly, utilization of home remedies to ease dental pain by Afghani refugees [15] can be explained as an impact of their cultural beliefs on their dental attitude. Furthermore, culture intermingled with religion seems to be very important among Muslim participants [15,22]. A longer stay in the host country influenced the care seeking behavior positively for most women [9,10], which explains the impacts of acculturation on oral health in this study group. However, only a few studies assessed this relation and included only women from Pakistan and Bangladesh. 

Fewer studies assessed Bhutanese [21] or Afghani refugees [15] even though migration has been common from this region [30]. The major ethnicities explored in-depth were Indian, Pakistani, and Bangladeshi. Sri Lankan, Nepalese, and Maldivian adults were not included in any study. To assess these large growing ethnicities in overcoming the dental health challenges in high-income countries, it is imperative to design not only larger studies but also more studies about these groups as they are increasingly making up more of the recent migration [32] compared to earlier South Asian migration patterns, which were predominately of Pakistani and Indian origin. Secondly, earlier migrants from these countries were educated and often came as skilled migrants whereas now due to poverty, war, and increasing refugee movements, the migrants are from mostly rural or small communities with low health literacy, education, and knowledge.

The main strength of this review is the number of studies that included a range of South Asian migrants, which was a result of our intense search strategy encompassing major electronic databases. The qualitative studies included were in-depth in assessing the beliefs and barriers of the South Asian migrants. Although we included studies that assessed clinical outcomes, we only focused on assessing the knowledge, attitudes, and practices. Another important strength of this review is that we assessed several key domains including knowledge, attitudes, beliefs, behaviors, and understandings, which makes our review unique.

The included studies varied in quality and some had their own methodological limitations such as not having assessed the impact of migration status on the experience of the participants or the duration of stay in the host country which would mean varying degrees of acculturation. Other limitations were that unpublished material, as well as those published in languages other than English, were not considered in this review. Furthermore, the migrant populations included in most studies were limited to either Indian, Pakistani, or Bangladeshi migrants with very few on Afghani and Bhutanese migrants. 

There was a dearth of information on other South Asian populations including Sri Lankan, Maldivian, and Nepalese migrants. There are large numbers of these populations already settled in Western countries and recent economic, social, political, or environmental push or pull factors have been drivers of the current high migration trends to Western countries [32]. In addition, some of the included studies were only on women with no males participating for comparison. However, the lack of literature is not a reflection on the quality of this paper, but rather the existing literature.

## 5. Conclusions

In summary, oral health literacy is low in adult South Asian migrants and prevention practice is strongly influenced by gender, religion, and traditional practices. If educational interventions were developed, they need to consider cultural diversity and religiosity for maximum effectiveness. Further in-depth studies are needed to explore the impact of acculturation on oral health, if any. Little is known about migrants from Afghanistan, Bhutan, the Maldives, and Sri Lanka, a rapidly growing population, and needs to be understood to better meet the oral health needs of these possible at-risk groups. As a start, targeted oral health educational strategies focusing on females within these communities may provide the necessary first step to improving oral health. 

## Figures and Tables

**Table 1:** Search terms used for the search strategy.

Search Terms
Oral health OR
Dental Health OR
Oral hygiene OR
Dental hygiene OR
Oral Care OR
Dental Care OR
AND
South Asia * OR
India * OR
Sri Lanka * OR
Nepal * OR
Bangladesh * OR
Bhutan * OR
Afghanistan * OR
Maldiv * OR
Pakistan * OR
AND
Migra * OR
Immigra * OR
Emmigra *OR
Ethnic * OR
Minorit *
AND
Knowledge OR
Attitude OR
Practice OR
Belief OR
Understanding OR
Behavior OR
Habit

* has been used as a wildcard symbol to broaden the search. Further hand searches were conducted using citations from included publications and related review papers.

**Table 2:** Summary characteristics table of qualitative studies.

First Author, Pub. Year (Ref.)	Study Population (Which Countries Included as South Asian)	The Country the Study Was Conducted in (South Asians Were Migrants or Ethnic Group Within This Region, State or Country) and Period of Data Collection	Number of Adults in the Sample. Stratified by South Asian Region and Age Group	Methodology	Aim of the Study	The Outcome That the Study Was Assessing and Whether They Stratified by Asian Status	Brief Description of Differences of What They Found Between the South Asian Groups Studied
Williams 1988 [24]	Bangladeshi and Pakistani as South Asians	London, April–June 1987	*N* = 100 total *N* = 50 Bangladeshi *N* = 50 Pakistani Age group—not mentioned	Interviews with 20 focus groups with 5 mothers in each.	To explore the ways to improve the consumption of dental services by Muslim mothers from Bangladesh and Pakistan.	Awareness regarding the availability of services, barriers to the use of services, and establish possible ways to improve the availability of services for Muslim mothers in the UK. Results not described according to the country of birth.	Muslim mothers were well aware of the services available. Presence of a symptom was a requisite for the majority of mothers to visit a dentist and less than half recognized the significance of a regular check-up. Visiting a dentist was not the priority for Muslim mothers. Fear, lack of trust, and communication difficulties were identified as potential barriers to the uptake of services. Preference for female dentists was highlighted by the mothers. Lack of required knowledge regarding oral health was prevalent in all the groups. An absence of cultural sensitivity was emphasized.
Newton 2001 [25]	Out of seven ethnic groups they included, Pakistani, Indian, and Bangladeshi as South Asians	South Thames region, UK	*N* =193 total *N* = 3 Pakistani groups *N* = 4 Indian groups *N* = 5 Bangladeshi groups. Age group—not mentioned	28 focus group interviews with each representing a particular ethnic group.	Identification of barriers for the utilization of dental services among various ethnic group residing in the UK through a qualitative approach.	Discussion and presentation of views around various pre-identified barriers such as language, trust, cost, anxiety, and cultural issues between various ethnic groups.	Language stated as a major barrier by nearly half of the participants from Bangladeshi, Pakistani, and Indian origin. Bangladeshi and Pakistani participants recommended that translation facilities should be available as one of the strategies for improving the uptake of services. Lack of trust for the dentist was cited as a major issue by Bangladeshi, Pakistani, and Indian participants. The cost was also stressed but to a lesser extent, Bangladeshi, Pakistani, and Indian participants demanded a change in the payment system. Preference for a woman dentist was observed among Bangladeshi women. Lack of cultural sensitivity by the dentist was mentioned by Indian participants. Concerns of hygiene were identified only by Pakistani and Chinese/Vietnamese groups.
Croucher 2006 [23]	Out of three, Indian and Bangladeshi as South Asians	East London, July–August 2001	*N* = 68 total *N* = 9 Indian *N* = 13 Bangladeshi Age group = 18–40	Rapid participatory approach, 12 focus groups who had in-depth discussions	To ascertain and compare the barriers for the use of dental services by adults in specific ethnic groups vs. the general population	Insights regarding the structure of dental care, barriers to use services, and proposals to improve access by the ethnic groups who conversed.	The long waiting list, dentist being overworked, and lengthy treatments were acknowledged by Indians whereas distance to access a dentist was acknowledged by Bangladeshi. Cost and lack of knowledge of average prices of various treatments were the other concerns specified by Indians. Dentist of the same gender was not a great requirement by an Indian woman as compared to a Bangladeshi woman. Recommendation of staff having training in local community languages was quoted by a Bangladeshi woman. Perception of “clean practice” was prevalent among the participants. Having whiskey was preferred than visiting a dentist by an Indian man to ease the dental pain.
Riggs 2014 [14]	Pakistani as South Asians	Australia	*N* = 115 total *N* = 3 focus groups (20) Pakistani. Individual interviews with 4 Pakistani women. Age group—not mentioned	Participatory research approach focus group (11) and individual (7) in-depth interviews.	To present the experiences detailed by Iraqi, Lebanese, and Pakistani women in dental service utilization for themselves and their children in Melbourne, Australia.	Iraqi, Lebanese, and Pakistani women were interviewed in depth for their experiences and barriers in accessing dental services in Melbourne. Participants were further probed for their oral health behaviors.	The majority did not access dentists for preventive purposes but only for treatment. Pakistani women preferred to pursue treatments in Pakistan as they believe the service would be provided by a qualified doctor at cheaper prices and there would be no language barriers. Pakistani perceive the restorative treatments as more expensive, so were more inclined to extractions. Lack of knowledge of the type of oral hygiene aid was evident as miswak was still used by some of the participants. Pakistani women felt being judged on the basis of their culture and country of origin. Halal certification was stated as one of the prerequisites for the health professional to treat them.
Lamb 2009 [15]	Afghanistan refugees as South Asians	Australia, July–August 2001.	*N* = 8 total Age group > 20 years	Semi-structured in-depth interviews	To describe the oral health understandings presented by the group of Afghan refugees.	Group of Afghan refugees were questioned for their views on oral health risk factors, the motivation for oral care, access to a dentist, pain management, and oral health education.	Numerous risk factors for oral health were acknowledged among refugees like smoking chelam, sucking naswar, breaking nuts, and stress of survival. Oral hygiene was stated as a requirement for religious purposes with some mentioned using miswak three times a day. Different oral hygiene aids were used by the study population such as salt, fingers, toothpaste, and toothbrush. Home remedies (cloves, aspirin yeast, takhak, salt water rinse) were preferred more to ease pain then to visit a dentist. Hazaras were also found to carry out extractions by themselves under unhygienic conditions. The existence of belief that the filling does not work was found.

**Table 3:** Summary characteristics table of quantitative studies.

First Author, Pub. Year (Ref.)	Study Population (Which Countries Included as South Asian)	The Country the Study Was Conducted in (South Asians Were Migrants or Ethnic Group Within This Region, State or Country) and Period of Data Collection	Number of Adults in the Sample. Stratified by South Asian Region and Age Group	Survey Instrument	Aim of the Study	The Outcome That the Study Was Assessing and Whether They Stratified by Asian Status	Brief Description of Differences of What They Found Between the South Asian Groups Studied
Arora 2017 [8]	Out of five groups, they included Indian and combined Pakistani/Bangladeshi as two groups as South Asians	UK, study conducted in 2009 to 2010	*N* = 10,435 total *N* = 272 Indians *N* = 165 Pakistani/Bangladeshi Age group > 16	Validated instrument Adult Dental Health Survey (ADHS)	To examine oral health differences among different ethnic groups. Really trying to ascertain whether the lifestyle factors and use of dental services contribute to the oral health disparities.	They conducted a logistic regression analysis of clinical outcomes. They presented descriptive tables on behaviors such as frequency of teeth cleaning, visits to dentist, and use of dental hygiene products by various ethnic groups. They also depicted the differences in the perception of oral health among different ethnic groups.	South Asians were less likely to consume sweets, cakes, and fizzy drinks but more likely to add sugar to hot drinks and this pattern was similar among all South Asian subgroups. A higher proportion (71.5%) of Pakistanis/Bangladeshis than Indians (64.2%) reported brushing twice a day. However, 20.4% of Pakistanis/Bangladeshis and 19.4% of Indians visit the dentist only if they have a symptom. Majority of South Asians do not use other oral hygiene products.
Robinson 2000 [13]	Out of seven groups, Pakistani, Indian, and Bangladeshi represented three groups as South Asians	South Thames, UK	*N* = 1113 total *N* = 123 Pakistani *N* = 190 Indian *N* = 78 Bangladeshi Age group > 16	Questionnaire	To assess the oral health status and its determinants among various ethnic groups.	Descriptive statistics of oral health-related behaviors such as daily cleaning of teeth, the frequency of visit to the dentist, and sugar intake, were stated according to various ethnic groups.	A higher percentage of Indians reported cleaning teeth daily (98.9%) and visiting the dentist annually (99.5%) followed by Bangladeshi and Pakistani. Sugar exposure was almost comparable among all the groups.
Taylor 1983 [20]	Indian, Pakistani, and Bangladeshi as South Asians	Britain	*N* = 231 total *N* = 109 adults *N* = 45 Bangladeshi *N* = 34 Indian *N* = 30 Pakistan Age group > 14	Questionnaire	To explore the number of aspects in regard to dental awareness, dietary patterns, and dental care amongst the Asian community.	Descriptive information of the population is provided according to various variables such as dietary patterns, tooth brushing, visits to a dentist, and dental awareness, and tabulated according to the country of origin.	Pakistani and Bangladeshi had high mean sugar intake score. Very few individuals among all the groups reported twice brushing as a habit. Majority of Pakistanis and Indians visited the dentist when in pain.
Qui 2003 [29]	Out of 8 groups, Asian Indians were only representing South Asians	The United States during 1997–2000	*N* = 110,844 total *N* = 798 Asian Indians Age group > 18	National Health Interview Surveys (NHISs)	To present the national estimates of dental service utilization by various ethnic groups.	Estimates of dental care utilization were provided for Asians and for Asian Indians. Furthermore, the percentage of Asians who had visited the dentist in the past year by various characteristics was also available, but not stratified for Asian Indians.	Asian Indians (8.1%) had never visited the dentist. Compared to other groups, d Asian Indians were least likely to visit the dentist.
Soh 1992 [30]	Out of three, Indians were only group of South Asians	Singapore	*N* = 446 total *N* = 34 Indian Age group > 18	Telephone interview survey	To examine the racial difference in knowledge of preventive measures for oral health and use of the preventive services.	Chi-squared test was used to assess the racial differences for the knowledge and the behaviors of oral health such as tooth brushing, flossing, regular check-up, fluoride, and dental sealants.	Importance of flossing was less evident for Indians (64.7%) compared to other groups. Dental check-ups were considered unnecessary for the majority of Indians (88.9%) with no dental care. All Indians were found to appreciate the benefits of effective brushing and had good knowledge of the role of fluoridated toothpaste in oral health.
Kavathe 2018 [16]	Out of four groups, Indian and Pakistani as South Asians	New York, USA	*N* = 169 total *N* = 165 Indian *N* = 4 Pakistani Age group > 18	A validated survey instrument adapted from the National Health Interview Survey, National Health and Nutrition examination survey, Behavioral Risk Factor Surveillance system, and New York City Community Health Survey.	To describe how oral health was identified as a priority for Sikh Asian Indian population by the United Sikhs through the community needs and resource assessment (2010) conducted for diabetes prevention. Furthermore, how they used it to develop a curriculum for the population.	Descriptive statistics were provided from community need and resource assessment to form the basis for oral health priority (2010) such as frequency of dental check-ups. Further, results of a descriptive study of oral conditions (2013) such as availability of dental insurance in the population and frequency of visiting the dentist were presented. Results not categorized by the place of birth.	According to community needs and resource assessment (2010), the majority (57%) never had a screening or check-up by the dentist. Descriptive study (2013), higher percentage (80.2%) were without dental insurance, a regular dentist (64.6%) or needed dental care (72.9%).
Jones 1987 [27]	Only Indian as South Asians	England	*N* = 231 total Age group = 15–59	Structured questionnaire	To determine the oral hygiene practices among migrant Asian females of Indian origin.	Oral hygiene practices such as agents used for tooth cleaning, interdental, mouth, and tongue cleaning were determined and categorized according to age group.	A vast number of females reported the use of toothpaste (68.4%) and a toothbrush (67.5%) with the majority in the age group (20–29 years). Few (17.3%) used their finger and 1.3% used datun. Only 6.9% were found using floss as an interdental aid. Tooth cleaning appeared in only 24.2% of the sample.
Selikowitz 1986 [9]	Only Pakistani as South Asians	Norway, 1982	*N* = 96 total Age group > 20	Structured questionnaire	To observe the pattern of utilization of dental services among Pakistani migrants with regards to migration variables.	Statistical analysis was employed (Chi-squared test) to investigate the differences in utilization of services by the population according to various variables such as the number of years in Norway, knowledge of causes of oral diseases, and belief about consequences of dental diseases.	Utilization of dental services was observed to be similar among all the age groups. Immigrants who lived in Norway for 1–6 years were found to use more services compared to those with more than 9 years in Norway, but the difference was not statistically significant. Those who believed (55.0%) that dental diseases are dangerous were in the category of high utilization of services compared to those who did not believe (*p* < 0.05).
Williams 1996 [10]	Only Bangladeshi as South Asians	West Yorkshire, UK	*N* = 246 total Age group > 25 years.	Interview	To assess the oral health status of Bangladeshi-born women and the relationship with various social, demographic, and behavioral variables.	Comparison of Bangladeshi women socio-demographic variables with oral hygiene practices categorized into traditional (finger, chewing stick, soot, tobacco powder), conventional (toothpaste and toothbrush) and combination category and further with dental visiting habits.	A large number of participants were observed to follow the traditional method of tooth cleaning and never visited a dentist. Years lived in the UK was found to be positively associated with dental attendance but not associated with practices. Traditional methods were more prevalent among the ones who never visited the dentist (*p* < 0.01).
Ghiabi 2013 [21]	Only Bhutanese as South Asians	Nova Scotia, Canada	*N* = 96 total *N* = 41 Bhutanese Age group = 18–67 years.	2008 Canadian Health Measures Survey (oral health module)	To describe the findings of oral health survey among the group of recent migrants and Bhutanese refugees in Canada.	Self-reported oral health, including the frequency of oral care such as tooth brushing, flossing, and dental visits were compared between Bhutanese refugees and other migrants in Canada.	Significant numbers of Bhutanese never floss (95.1%) and visit the dentist only in an emergency (53.7%). None of the refugees had dental insurance, however, a higher proportion was found to brush twice a day.
Cruz 2009 [28]	The only group as South Asians were Indian	New York, 1996–2001	*N* = 1318 total *N* = 196 Indian Age group = 18–65 years	Validated structured questionnaire	To ascertain the relationship of oral health of immigrants with various demographic and risk factors.	Descriptive analysis of the study population sub-classified by ethnic group (Asian Indian) presented with regard to frequency of brushing, flossing, visiting a dentist, length of stay in the USA, oral health knowledge, and attitude score.	About 84.7% Asian Indians were described to have dental insurance, daily brushing practice (95.9%), and high knowledge and attitude score (58.7% and 79.1%, respectively). Around 27% visited the dentist once a year.
Kay 1990 [22]	Indian were only group as South Asians	Glasgow, UK	*N* = 69 total Age group = 18–68 years.	Semi-structured questionnaire	To explore the knowledge, attitude, and behavior with regards to oral health among Asians residing in Glasgow.	Sample assessed for their knowledge (steps to take to reduce caries, deleterious effects of sweets on teeth and related to fluoride), attitude (reasons, cost, and frequency to visit dentist) and behavior (diet, oral hygiene, and dental health).	Harmful effects of sweets were appreciated by 64% of the sample whereas 66% consume the cariogenic diet. Knowledge in relation to fluoride was low (63%) and the population only received fluoride through the paste. All respondents acknowledged the importance of oral health but the majority (48%) mentioned cost as a potential barrier and 44 participants attended the dentist when in trouble. Around 41% of the population was found to brush twice and 68% rinsed their mouth after every meal.

## Appendix A

**Table A1:** PRISMA checklist.

Section/Topic	^#^	Checklist Item	Reported on Page ^#^
Title	1	Identify the report as a systematic review, meta-analysis, or both.	1
Abstract			
Structured summary	2	Provide a structured summary including, as applicable: background; objectives; data sources; study eligibility criteria, participants, and interventions; study appraisal and synthesis methods; results; limitations; conclusions and implications of key findings; systematic review registration number.	1
Introduction			
Rationale	3	Describe the rationale for the review in the context of what is already known.	1 and 2
Objectives	4	Provide an explicit statement of questions being addressed with reference to participants, interventions, comparisons, outcomes, and study design (PICOS).	2
Methods			
Protocol and registration	5	Indicate if a review protocol exists, if and where it can be accessed (e.g., Web address), and, if available, provide registration information including registration number.	2
Eligibility criteria	6	Specify study characteristics (e.g., PICOS, length of follow-up) and report characteristics (e.g., years considered, language, publication status) used as criteria for eligibility, giving rationale.	2 and 3
Information sources	7	Describe all information sources (e.g., databases with dates of coverage, contact with study authors to identify additional studies) in the search and date last searched.	2 and 3
Search	8	Present full electronic search strategy for at least one database, including any limits used, such that it could be repeated.	2 and 3
Study selection	9	State the process for selecting studies (i.e., screening, eligibility, included in systematic review, and, if applicable, included in the meta-analysis).	2 and 3
Data collection process	10	Describe method of data extraction from reports (e.g., piloted forms, independently, in duplicate) and any processes for obtaining and confirming data from investigators.	3
Data items	11	List and define all variables for which data were sought (e.g., PICOS, funding sources) and any assumptions and simplifications made.	3
Risk of bias in individual studies	12	Describe methods used for assessing risk of bias of individual studies (including specification of whether this was done at the study or outcome level), and how this information is to be used in any data synthesis.	3
Summary measures	13	State the principal summary measures (e.g., risk ratio, difference in means).	3
Synthesis of results	14	Describe the methods of handling data and combining results of studies, if done, including measures of consistency (e.g., I^2^) for each meta-analysis.	-
Risk of bias across studies	15	Specify any assessment of risk of bias that may affect the cumulative evidence (e.g., publication bias, selective reporting within studies).	3 and 4
Additional analyses	16	Describe methods of additional analyses (e.g., sensitivity or subgroup analyses, meta-regression), if done, indicating which were pre-specified.	-
Results			
Study selection	17	Give numbers of studies screened, assessed for eligibility, and included in the review, with reasons for exclusions at each stage, ideally with a flow diagram.	4
Study characteristics	18	For each study, present characteristics for which data were extracted (e.g., study size, PICOS, follow-up period) and provide the citations.	6–9, 10–16
Risk of bias within studies	19	Present data on risk of bias of each study and, if available, any outcome level assessment (see item 12).	20 and 21
Results of individual studies	20	For all outcomes considered (benefits or harms), present for each study: (a) simple summary data for each intervention group and (b) effect estimates and confidence intervals, ideally with a forest plot.	6–9, 10–16
Synthesis of results	21	Present results of each meta-analysis done, including confidence intervals and measures of consistency.	10,16
Risk of bias across studies	22	Present results of any assessment of risk of bias across studies (see item 15).	20 and 21
Additional analysis	23	Give results of additional analyses, if done (e.g., sensitivity or subgroup analyses, meta-regression (see item 16)).	-
Discussion			
Summary of evidence	24	Summarize the main findings including the strength of evidence for each main outcome; consider their relevance to key groups (e.g., healthcare providers, users, and policy makers).	17–18
Limitations	25	Discuss limitations at study and outcome level (e.g., risk of bias), and at review-level (e.g., incomplete retrieval of identified research, reporting bias).	19
Conclusions	26	Provide a general interpretation of the results in the context of other evidence, and implications for future research.	19
Funding	27	Describe sources of funding for the systematic review and other support (e.g., supply of data); role of funders for the systematic review.	19

^#^ Number Sign.

**Table A2:** Quality assessment of qualitative studies [18].

Study Assessment Question	Williams and Gelbier [24]	Newton et al. [25]	Croucher and Sohanpal [23]	Riggs et al. [14]	Lamb et al. [15]
Research design					
1. Are the study’s purpose and research aims clearly stated?	Yes (1)	Yes (1)	Yes (1)	Yes (1)	Yes (1)
2. Are the qualitative methods of inquiry appropriate for the study aims?	Yes (1)	Yes (1)	Yes (1)	Yes (1)	Yes (1)
3. Did the authors discuss why they decided to use qualitative methods?	No (0)	Yes (1)	Yes (1)	Yes (1)	Yes (1)
Sampling					
4. Is participant selection clearly described and appropriate?	Yes (1)	No (0)	Yes (1)	Yes (1)	Yes (1)
5. Is the sample size discussed and justified?	No (0)	No (0)	No (0)	No (0)	No (0)
Data collection					
6. Are data collection methods clearly described and justified?	Yes (1)	Yes (1)	Yes (1)	Yes (1)	Yes (1)
7. Are the methods appropriate given the study aims and research questions?	Yes (1)	Yes (1)	Yes (1)	Yes (1)	Yes (1)
Data analysis					
8. Is the analytic process clearly described?	No (0)	Yes (1)	Yes (1)	Yes (1)	Yes (1)
9. Were all relevant data taken into account?	Yes (1)	Yes (1)	Yes (1)	Yes (1)	Yes (1)
10. Did the authors consider/discuss contradictory evidence and data?	No (0)	Yes (1)	Yes (1)	Yes (1)	Yes (1)
11. Did the study include triangulation (namely, comparison of different sources of data re: the same issue)?	No (0)	No (0)	Yes (1)	No (0)	No (0)
12. Did triangulation produce convergent conclusions?	No (0)	No (0)	Yes (1)	No (0)	N/A
13. If “no,” was this adequately explained?	N/A	Yes (1)	N/A	Yes (1)	No (0)
14. Were the study’s findings generated by more than one analyst?	No (0)	Yes (1)	Yes (1)	No (0)	Yes (1)
Findings/Results					
15. Is there a clear statement of the findings?	Yes (1)	Yes (1)	Yes (1)	Yes (1)	Yes (1)
16. Are the study’s findings discussed in terms of their relation to the research questions posed?	Yes (1)	Yes (1)	Yes (1)	Yes (1)	Yes (1)
17. Do the findings appear credible?	Yes (1)	Yes (1)	Yes (1)	Yes (1)	Yes (1)
18. Are sufficient data presented to support the findings?	Yes (1)	Yes (1)	Yes (1)	Yes (1)	Yes (1)
19. Are potential researcher biases taken into account?	No (0)	No (0)	No (0)	No (0)	No (0)
20. Are conclusions explicitly linked with exhibits of data?	Yes (1)	Yes (1)	Yes (1)	Yes (1)	Yes (1)
Research value					
21. Do the study’s findings contribute to the current knowledge base?	Yes (1)	Yes (1)	Yes (1)	Yes (1)	Yes (1)
22. Can the findings reasonably be expected to inform current practices or policies?	Yes (1)	Yes (1)	Yes (1)	Yes (1)	Yes (1)
23. Are these contributions discussed by the authors?	Yes (1)	Yes (1)	Yes (1)	Yes (1)	Yes (1)
24. Did the authors identify new research areas?	No (0)	Yes (1)	Yes (1)	Yes (1)	Yes (1)
25. Did the authors discuss how the research findings could be used and for what populations?	Yes (1)	Yes (1)	Yes (1)	Yes (1)	Yes (1)
26. Was enough descriptive detail included to allow readers to make their own judgments about potential transferability to other settings?	Yes (1)	Yes (1)	Yes (1)	Yes (1)	Yes (1)

**Table A3:** Quality assessment for quantitative studies [19].

Study Assessment Question	Arora et al. [8]	Robinson et al. [13]	Taylor [20]	Qui and Ni [29]	Soh [30]	Kavathe et al. [16]	Jones et al. [27]	Selikowitz and Holst [9]	Williams et al. [10]	Ghiabi et al. [21]	Cruz et al. [28]	Kay et al. [22]
1. Is the research question clearly stated?	Yes	Yes	Yes	Yes	Yes	Yes	Yes	Yes	Yes	Yes	Yes	Yes
2. Are the criteria for selecting the sample clearly defined?	Yes	Yes	No	Yes	Yes	Yes	Yes	Yes	Yes	Yes	Yes	Yes
3. Is the method of recruitment clear?	Yes	Yes	Yes	Yes	No	Yes	Yes	Yes	Yes	Yes	Yes	Yes
4. Are the characteristics of the sample adequately defined?	Yes	Yes	No	Yes	No	Yes	No	Yes	Yes	Yes	Yes	Yes
5. Is the final sample adequate and appropriate?	No	Yes	No	Yes	No	No	Yes	No	Yes	No	Yes	Yes
6. Was the method for collecting data adequately described?	Yes	Yes	No	Yes	Yes	Yes	Yes	Yes	Yes	Yes	Yes	Yes
7. Was the data collected systematically?	Yes	Yes	U/D	Yes	Yes	Yes	Yes	Yes	Yes	Yes	Yes	Yes
8. Was the relationship between the researcher and the participant explicit?	Yes	Yes	Yes	Yes	Yes	Yes	Yes	Yes	Yes	Yes	Yes	Yes
9. Were the methods used in the data analysis appropriate and designed to minimize bias?	Yes	Yes	No	Yes	No	Yes	No	Yes	Yes	N/A	Yes	N/A
10. Is evidence provided in support of the analysis?	Yes	Yes.	Yes	Yes	Yes	Yes	No	Yes	Yes	Yes	Yes	Yes
11. Is there evidence of efforts to establish validity?	Yes	Yes	No	Yes	No	No	No	No	Yes	Yes	Yes	N/A
12. Were the conclusions drawn appropriate given the results?	Yes	Yes	No	Yes	Yes	Yes	No	Yes	Yes	Yes	Yes	Yes

## References

[1] Jin L.J., Lamster I.B., Greenspan J.S., Pitts N.B., Scully C., Warnakulasuriya S. (2015). Global burden of oral diseases: Emerging concepts, management and interplay with systemic health. Oral Dis..

[2] Isola G., Cicciu M., Fiorillo L., Matarese G. (2017). Association between odontoma and impacted teeth. J. Craniofac. Surg..

[3] Mattos M.G., Fernandez C.A., Masterson D., Maia L.C., Neves A.A. (2019). Is the caregivers’ oral health related to dental caries in children or adolescents? A systematic review. Clin. Oral Investig..

[4] Petersen P.E. (2008). The World Oral Health report 2003: Continuous improvement of oral health in the 21st century—The approach of the WHO Global Oral Health Programme. Community Dent. Oral Epidemiol..

[5] Isola G., Matarese G., Cordasco G., Rotondo F., Crupi A., Ramaglia L. (2015). Anticoagulant therapy in patients undergoing dental interventions: A critical review of the literature and current perspectives. Minerva Stomatol..

[6] Kandelman D., Arpin S., Baez R.J., Baehni P.C., Petersen P.E. (2012). Oral health care systems in developing and developed countries. Periodontol. 2000.

[7] World Health Organization What Is the Burden of Oral Disease?. http://www.who.int/oral_health/disease_burden/global/en/.

[8] Arora G., Mackay D.F., Conway D.I., Pell J.P. (2017). Ethnic differences in oral health and use of dental services: Cross-sectional study using the 2009 adult dental health survey. BMC Oral Health.

[9] Selikowitz H.S., Holst D. (1986). Dental health behavior in a migrant perspective: Use of dental services of Pakistani immigrants in Norway. Community Dent. Oral Epidemiol..

[10] Williams S.A., Summers R.M., Ahmed I.A., Prendergast M.J. (1996). Caries experience, tooth loss and oral health-related behaviours among Bangladeshi women resident in West Yorkshire, UK. Community Dent. Health.

[11] U.S. Census Bureau (2018). American Community Survey.

[12] Australian Bureau of Statistics (2018). 3412.0—Migration, Australia, 2016–17.

[13] Robinson P.G., Bhavnani V., Khan F.A., Newton T., Pitt J., Thorogood N., Gelbier S., Gibbons D. (2000). Dental caries and treatment experience of adults from minority ethnic communities living in the South Thames Region, UK. Community Dent. Health.

[14] Riggs E., Gussy M., Gibbs L., Van Gemert C., Waters E., Kilpatrick N. (2014). Hard to reach communities or hard to access services? Migrant mothers’ experiences of dental services. Aust. Dent. J..

[15] Lamb C.E.F., Whelan A.K., Michaels C. (2009). Refugees and oral health: Lessons learned from stories of Hazara refugees. Aust. Health Rev..

[16] Kavathe R., Islam N., Zanowiak J., Wyatt L., Singh H., Northridge M.E. (2018). Building capacity in the Sikh Asian Indian community to lead participatory oral health projects. Prog. Community Health Partnersh..

[17] Moher D., Liberati A., Tetzlaff J., Altman D.G., The PRISMA Group (2009). Preferred Reporting Items for Sytematic Reviews and Meta-Analyses: The PRISMA Statement. PLoS Med..

[18] Kennelly J., Handler A., Kennelly J., Peacock N. (2011). Methodological approach to assessing the evidence. Reducing Racial/Ethnic Disparities in Reproductive and Perinatal Outcomes: The Evidence from Population-Based Interventions.

[19] Downs S.H., Black N. (1998). The feasibility of creating a checklist for the assessment of the methodological quality both of randomised and non-randomised studies of health care interventions. J. Epidemiol. Community Health.

[20] Taylor W. (1983). Dietary patterns, dental awareness and dental caries in the Asian community. Dent. Health.

[21] Ghiabi E., Matthews D.C., Brillant M.S. (2014). The oral health status of recent immigrants and refugees in Nova Scotia, Canada. J. Immigrant Minority Health.

[22] Kay E.J., Shaikh I., Bhopal R.S. (1990). Dental knowledge, beliefs, attitudes and behaviour of the Asian community in Glasgow. Health Bull..

[23] Croucher R., Sohanpal R. (2006). Improving access to dental care in East London’s ethnic minority groups: Community based, qualitative study. Community Dent. Health.

[24] Williams S.A., Gelbier S. (1988). Access to dental health? An ethnic minority perspective of the dental services. Health Educ. J..

[25] Newton J.T., Thorogood N., Bhavnani V., Pitt J., Gibbons D.E., Gelbier S. (2001). Barriers to the use of dental services by individuals from minority ethnic communities living in the United Kingdom: Findings from focus groups. Primary Dent. Care.

[26] Ahmed F., Abel G.A., Lloyd C.E., Burt J., Roland M. (2015). Does the availability of a South Asian language in practices improve reports of doctor-patient communication from South Asian patients? Cross sectional analysis of a national patient survey in English general practices. BMC Fam. Pract..

[27] Jones C.V., Srivastava R.P., Walsh T.F. (1987). Oral hygiene practices in a group of Asian females. Dent. Health.

[28] Cruz G.D., Chen Y., Salazar C.R., Le Geros R.Z. (2009). The association of immigration and acculturation attributes with oral health among immigrants in New York city. Am. J. Public Health.

[29] Qiu Y., Ni H. (2003). Utilization of dental care services by Asians and native Hawaiian or other Pacific Islanders: United States, 1997–2000. Adv. Data.

[30] Soh G. (1992). Racial differences in perception of oral health and oral health behaviours in Singapore. Int. Dent. J..

[31] Riggs E., Gussy M., Gibbs L., Gemert C., Waters E., Priest N., Watt R., Renzaho A.M.N., Kilpatrick N. (2014). Assessing the cultural competence of oral health research conducted with migrant children. Community Dent. Oral Epidemiol..

[32] The United Nations (2016). International Migration Report 2015.

[33] Ram S. (1987). Indians in England: Why did they emigrate?. Popul. Geogr..

[34] Office of National Statistics (2013). 2011 Census: Key Statistics and Quick Statistics for Local Authorities in the United Kingdom.

[35] Lukacs John R. (2011). Gender differences in oral health in South Asia: Metadata imply multifactorial biological and cultural causes. Am. J. Hum. Biol..

